# Manufacture of patient-specific vascular replicas for endovascular simulation using fast, low-cost method

**DOI:** 10.1038/srep39168

**Published:** 2016-12-15

**Authors:** Naoki Kaneko, Toshihiro Mashiko, Taihei Ohnishi, Makoto Ohta, Katsunari Namba, Eiju Watanabe, Kensuke Kawai

**Affiliations:** 1 Jichi Medical University, Department of Neurosurgery, Shimotsuke, Japan; 2 Tohoku University, Institute of Fluid Science, Sendai, Japan; 3 Jichi Medical University, Department of Endovascular Surgery, Shimotsuke, Japan

## Abstract

Patient-specific vascular replicas are essential to the simulation of endovascular treatment or for vascular research. The inside of silicone replica is required to be smooth for manipulating interventional devices without resistance. In this report, we demonstrate the fabrication of patient-specific silicone vessels with a low-cost desktop 3D printer. We show that the surface of an acrylonitrile butadiene styrene (ABS) model printed by the 3D printer can be smoothed by a single dipping in ABS solvent in a time-dependent manner, where a short dip has less effect on the shape of the model. The vascular mold is coated with transparent silicone and then the ABS mold is dissolved after the silicone is cured. Interventional devices can pass through the inside of the smoothed silicone vessel with lower pushing force compared to the vessel without smoothing. The material cost and time required to fabricate the silicone vessel is about USD $2 and 24 h, which is much lower than the current fabrication methods. This fast and low-cost method offers the possibility of testing strategies before attempting particularly difficult cases, while improving the training of endovascular therapy, enabling the trialing of new devices, and broadening the scope of vascular research.

Minimally invasive endovascular procedures have been replacing traditional open surgeries as the primary therapeutic modality for vascular diseases from the head to the lower limbs^1^,^2^,^3^,^4^. This progress has been facilitated by the development of novel devices and techniques as a result of continuously evolving materials science and technology. A prominent example is the endovascular treatment of cerebral aneurysms^5^. Therapeutic devices, such as platinum coils, stents, and flow diverters, are currently available^6^,^7^,^8^, and new options are being developed. When devising a treatment strategy, it is crucial to have access to a range of approaches and a combination of devices and techniques prior to the treatment, in order to attain a safe and good outcome. Images obtained from computed tomography angiography (CTA) or three-dimensional rotational angiography (3DRA) are generally used for developing strategies. However, it is difficult to predict the actual behavior of devices by using 3D images on a screen because the cerebral vessels are tortuous and multiple devices can interfere with one another.

Vascular replicas of cerebral aneurysms from real patient data have been adopted for the evaluation of new devices, hemodynamic analysis, or training^9^,^10^,^11^. However, it has not been widely utilized as a routine clinical process due to the considerable labor, time for the fabrication (more than seven days) and the high prototyping cost (USD $600). In addition, the price of 3D printers was more than USD $50,000^10^,^12^. Therefore, the application of a vascular replica to pre-operative simulations for each treatment was impractical. We previously reported on a pre-surgical clipping simulation system that used a vascular replica made using a low-cost ($1,300) acrylonitrile butadiene styrene (ABS) printer^13^. This quick and low-cost method is useful for aneurysmal clipping simulation but is not suitable for endovascular treatment due to difficulty with manipulating interventional devices in the silicone replica.

An ABS 3D printer utilizes a fused filament fabrication (FFF) rapid prototyping system, in which a thermoplastic filament is melted and positioned layer upon layer, working from the bottom up. The action of the FFF system creates stair-like layers with about 150 μm on the surface of the vascular mold, which causes resistance to manipulation and unclarity of silicone. Although smoothing by sanding of the printed object is popular, it is time-consuming and labor-intensive, and can affect the conformity of the object.

In this article, we describe a new low-cost and rapid method for manufacturing patient-specific silicone replicas for the endovascular simulation of cerebral aneurysms. Especially, we evaluate the new method as a means of smoothing the surface of an ABS plastic object after printing.

## Results

**Figure 1:**
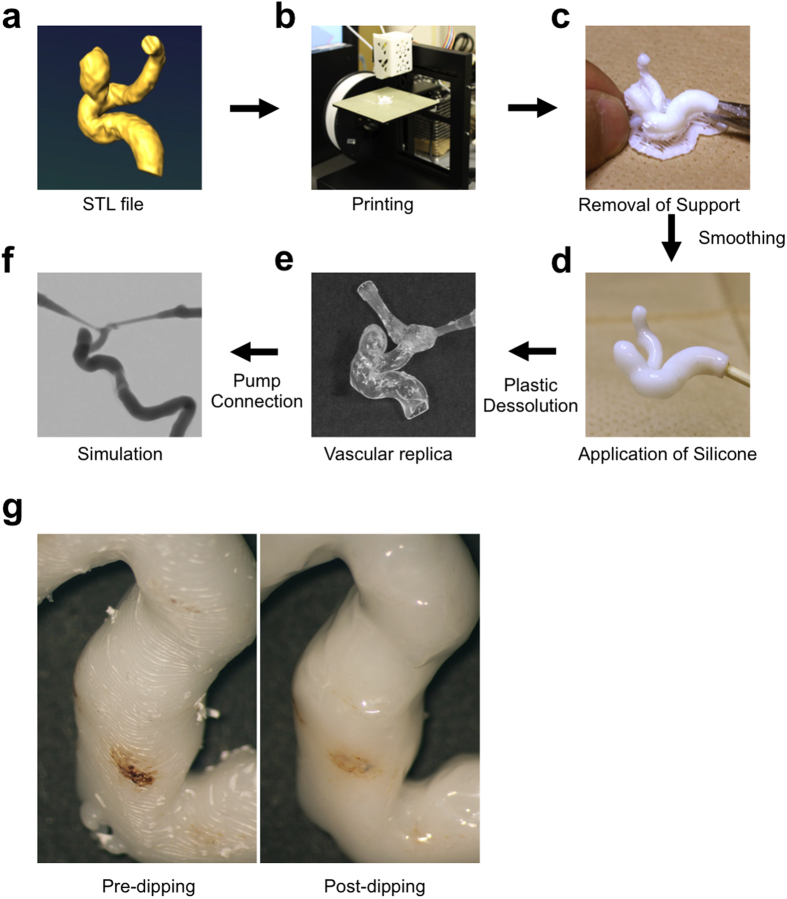
Manufacturing process of patient-specific vessel simulator. (**a**) DICOM data is obtained by CT, MRA, or rotational angiography. The data is converted to an STL file. (**b**) The STL file is exported and the ABS vascular model is printed using the 3D printer. (**c**) The ABS solid model and supports are removed with nippers. The surface is smoothed by dipping into ABS solvent and then dried. (**d**) The solid ABS model is coated with liquid silicone. (**e**) The ABS is dissolved in acetone after the silicone solidifies. (**f**) The silicone vessel is connected to a circulation pump and the simulation is performed. (**g**) The surface of the ABS vessel model is smoothed by ABS solvent. Left: Before dipping in the ABS solvent, Right: After dipping in the ABS solvent.

First, we modified our previous method^13^ of fabricating a silicone vessel for the simulation of endovascular treatment (Fig. 1a–f). We used transparent silicone to visualize the interventional devices inside the vessel replica. After the silicone had been cured, the vascular casting mold was dissolved by acetone that attacks the ABS stronger than xylene. Although there are several methods available for polishing the surface of ABS plastic^14^,^15^,^16^, those methods require considerable time and effort. We found that the ABS solvent, eSolve (Kaneko Chemical, Saitama, Japan) which is a halogen alkylate, produces a smoothing effect after a single dipping (Fig. 1g).

**Figure 2:**
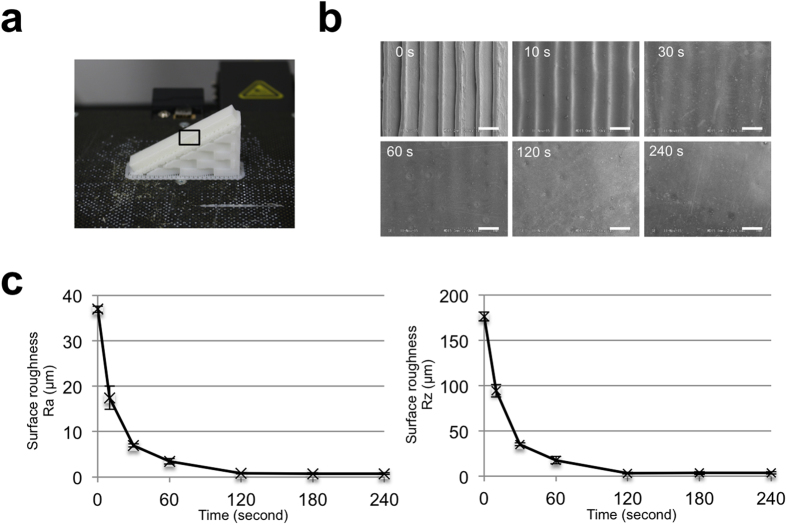
The smoothing effect of the ABS solvent on the surface of ABS plastic. (**a**) ABS plastic bar printed with layers at 30° angle. (**b**) Image of scanning electron microscope without dipping (0 s) and after dipping for the indicated duration. Scale bars are 500 μm. (**c**) Roughness on the surface of plastic bar without dipping and after dipping for the indicated duration. Data are mean ± standard deviation, n = 7.

We first sought to identify the smoothing effect of the ABS solvent on the surface of printed ABS plastic with stair-like layers with about 150 μm. To this end, we first examined the surface smoothness of ABS plastic by scanning-electron microscopy (SEM) after dipping them in ABS solvent for different durations. ABS plastic bars were printed with 30° angles because obliquely printed objects exhibit more evident stair-like layers than vertically or horizontally printed objects (Fig. 2a). SEM analysis showed that the surface of the ABS object that had not been dipped has clear stair-like steps (Fig. 2b). Dipping in the ABS solvent smoothed the surface of the plastic in a time-dependent manner. After only 10 s in the solvent, the edges of the stair-like steps were smoothed and the tiny gaps between the adjacent layers disappeared. Flat surfaces were obtained after dipping for 120 s.

We next quantified the surface roughness Ra and Rz by using a surface roughness measuring machine. Ra is the arithmetic mean of the absolute values and Rz is the average of the five highest peaks and lowest valleys within the sampling length. The application of ABS solvent to ABS plastic significantly decreased the surface roughness in a time-dependent manner (Fig. 2c), reaching a minimum at 120 s, which is consistent with the SEM observations. These findings demonstrate that a simple single dip in eSolve ABS solvent smooths the surface of the ABS plastic within 2 min.

**Figure 3:**
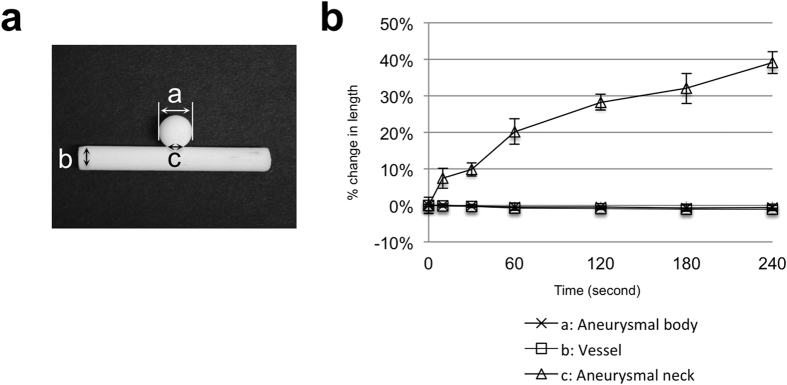
The effect of the ABS solvent on the diameter of the vascular model. (**a**) Photograph of vascular model with aneurysm. (**b**) Relationship between duration of dipping in ABS solvent and diameter. Data are mean ± standard deviation, n = 7.

There is the possibility that the ABS solvent could affect the conformity of the ABS plastic model, which would lead to the vascular replica differing from the patient data. To this end, we investigated the impact on the conformity of the vascular model after the dipping. Vascular ABS models of an aneurysm were manufactured (Fig. 3a) and dipped for different durations. The length of the aneurysmal of each specimen was measured using a digital micrometer (Mitutoyo Corp, Kawasaki, Japan). The application of the ABS solvent did not noticeably alter the diameter of the vessel or aneurysmal body, but significantly increased the neck length in a time-dependent manner (Fig. 3b). This was caused by the collection of fluid due to the surface tension that occurs in the tiny gap between a vessel and the aneurysmal body around the aneurysmal neck. The change in the neck diameter over 10 to 30 s is minimal, and the collection of ABS solvent after dipping was easily removed by a blow of a hair drier and with the sharp end of a small needle. The results indicate that dipping for more than 30 s increases the diameter of the neck but dipping for a relatively short duration of no more than 30 s has little effect on the diameter.

**Figure 4:**
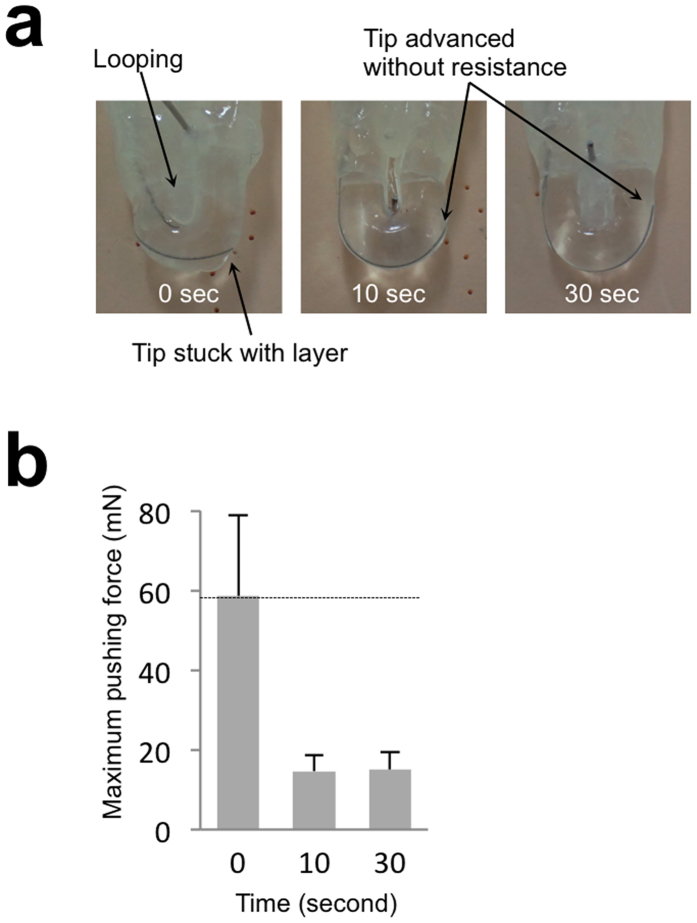
The effect of the smoothing on the movement of the microguidewire in the silicone vessel. (**a**) Photographs of U-shaped silicone vessel. In the silicone vessel without smoothing, the tip of the micro catheter stuck in the layers and looping was observed. With a short-duration smoothing process, the microguidewire can be propelled through the silicone vessel without becoming stuck. (**b**) Relationship between duration of dipping in ABS solvent and maximum pushing force. Data are mean ± standard deviation, n = 7.

To determine whether a treatment device can smoothly pass through the silicone vascular replica made from smoothed ABS plastic, we investigated the amount of force necessary to propel a microguidewire through the vessel replica. U-shaped silicon vessels were made from U-shaped plastic objects that were smoothed for different durations. The microguidewire tip was placed inside the U-shape silicone tube and was held by the grip of a force measuring machine (Fig. 4a). The grip was applied and the force was continuously measured until the tip of the microguidewire reached the other end of the U-shaped tube or until the grip displacement reached 20 mm. The maximum force required was significantly reduced by the smoothing of the ABS, even for as little as 10 s, compared to a tube made from ABS that had not been smoothed (Fig. 4b). In addition, the tip of the wire became stuck on its way to the end, thus forming a loop. This looping was observed in all seven trials although the point at which the wire became stuck was different every time, indicating that unsmoothed silicone is not suitable for simulation. On the other hand, the tip reached the end of the U-shaped tube without becoming stuck in all of trials with the silicon that had been smoothed for 10 or 30 s. These findings suggest that short-duration smoothing by ABS solvent reduces the friction between the silicone vessel and an endovascular device.

**Figure 5:**
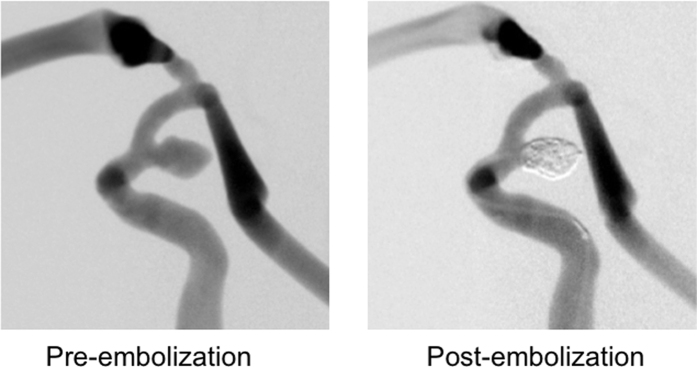
Digital subtraction angiograms of the silicone replica with internal carotid aneurysm. Left: Before coil embolization, Right: After coil embolization.

Lastly, to determine whether the manufactured silicone vessel can be applied to the simulation of endovascular treatment, we performed intrasaccular coiling in the smoothed aneurysmal model. A vessel mold with an internal carotid aneurysm was smoothed for 30 s and a silicone vessel was fabricated. The material cost and time required to fabricate the silicone vessel was about $2 and 24 h, which is much lower than the current fabrication methods. The range of the wall thickness was between about 420 and 680 μm. The replica was connected to a tube with a circulation pump and a water/surfactant mixture was circulated inside the tube. Endovascular coiling was performed using the actual devices that are used in practice (Fig. 5). We found that the sensation of manipulating the devices in the silicone replica is quite similar to that experienced in actual treatments. These findings show that a vascular replica that has been smoothed by simple dipping in ABS solvent is capable of being applied to the simulation of endovascular treatments.

## Discussion

In this study, we demonstrated that the short duration dipping of a printed vascular mold in an ABS solvent effectively smoothed the surface of the mold with a minimum change in the conformity of the vascular replica. In addition, we showed that the smoothing effect enabled the advancement of the endovascular device, and thus interventional simulation in the silicone vessel fabricated with our fast and low-cost method.

Silicone itself is known to have a high level of friction. Some attempts to minimize this friction have been reported^14^,^17^. Polyvinyl alcohol-hydrogel (PVAH) has a lower level of friction, but has a much shorter life than a silicone models^9^,^17^. This drawback of PVAH results from the instability against dryness and high temperature. Therefore, PVAH vessel replicas should be stored in cooled water and kept wet during endovascular simulations to avoid the deformation. On the other hand, silicone replicas can be stored at room temperature for a long time because of thermal and desiccation tolerance. Indeed, the material used for vascular replicas in most research is silicone, not PVAH^9^. In the present study, smoothing by a single dipping of an ABS mold removed the stair-like steps and allowed the passage of an endovascular device in a silicone wall without resistance. These findings suggest that our simple smoothing method is effective in decreasing the friction of silicone wall enough for performing an endovascular simulation, and that special coating of silicone for lowering friction is not indispensable.

In the literature, there are several studies that address the fabrication of a vascular replica^10^,^11^,^12^,^14^,^17^,^18^,^19^. The majority of these studies used ultraviolet-curing resin. Although the layer height that can be achieved using this material is thinner than is possible with an ABS printer, it is difficult to dissolve the vascular mold once the silicone has been cured. Therefore, the fabrication of a replica using this material incurs multiple cumbersome steps from negative molding through to positive wax casting. Wetzel *et al*. reported on a simple method in which a wax vascular cast was directly fabricated using a 3D printer, and then the wax was melted after the silicone had been cured^12^. However, a printed wax object is too fragile to smooth or use to form a replica. In our method, ABS can be used for repeated printing with a low-cost printer, after which it is smoothed by single short-term dipping in ABS solvent and dissolved by acetone after outer silicone is cured. Therefore, an ABS printer provides a cost effective and good means of fabricating a silicone vessel for endovascular simulation.

At present, there are several commercial silicone models available. While these models are useful for the training in endovascular intervention to improve hand-eye coordination, preoperative simulations of endovascular treatment would not be impractical due to high cost (more than USD $600) and long time (more than 7 days) for fabricating a patient-specific replica. Our method significantly reduced the material cost and time to about USD $2 and 24 h, which makes it possible to perform a preoperative simulation before a real endovascular treatment especially for a difficult case. In addition, our method has a potential to improve the previous models by friction-lowering effect on the artificial vessels, while the manufacturing methods of commercial models are undisclosed.

Although there have been some reports that address the polishing of the surface of ABS^14^,^15^,^16^, only a small number of these reports have confirmed the efficacy of smoothing. Recently, a report stated that acetone vapor provides an efficient means of smoothing the surface of the ABS^15^. However, the flammability of acetone makes it dangerous, especially when it is heated. In this experiment, we used an ABS solvent that is not designated as a dangerous material in Japan, and we confirmed that the surface of a silicone vessel could be polished through the application of an easy dipping method by electron microscopy and roughness. After we developed this method, we found that interventional devices can be slid inside the smoothed silicone vascular replica. This result indicates that the stair-like layers resulting from the printing process have a greater effect than the friction of the silicone.

Our technique does have limitations. The wall thickness is not uniform because we spread the silicone liquid manually. This non-uniform thickness might cause irregular expansion of the wall in experiments with pulsatile flow. However, given that our goal is to establish the method for manufacturing vascular replicas for endovascular simulation, this is not important because the non-uniform thickness of the wall would not make significant difference in the movement of interventional devices in the silicone vessels. Another limitation is that the content of the ABS filament used for the 3D printing process may differ depending on the manufacture, which affects the dissolution by the ABS solvent, although we confirmed that the ABS filaments from some manufactures could be smoothed by dipping in the ABS solvent.

In conclusion, we have developed a new method for fabricating patient-based vessel replicas for the endovascular treatment. The lower cost and speed of the method make it possible to perform case-based pre-operative simulation and training, as well as the trial of new devices and the promotion of endovascular research.

## Materials and Methods

### Model preparation

Autodesk’s Fusion 360 CAD program was used to design rectangular specimens for roughness measurement, as well as vascular models with aneurysms or U-shaped bends. A 3D solid vascular mold was formed using a 3D Printer (UP! Plus2; OPT, Tokyo, Japan). The cost of the printer was about $1,300. The thickness of each layer was set to 150 μm. The base and supports were manually removed after the model had been removed from the platform. The vascular mold made of ABS was immersed in ABS solvent for a prescribed time to smooth out the surface of the stair-like layers.

### Silicone vascular replica

The smoothed vascular mold was dried in air. The transparent silicone was degassed using a vacuum pump at room temperature. The vascular mold was coated with silicone and then heat-cured 60 °C for 30 min. The entire mold was immersed in acetone for 4 to 12 h to dissolve the ABS. The silicone vascular replica was washed with acetone several times and then dried. The silicone replica was connected to a silicone tube with a peristaltic pump (WPX1; Welco, Tokyo, Japan) to enable the simulation of endovascular treatment.

### Electron Microscopic Observation

The surfaces of ABS plastics were coated with platinum-palladium using an ion-sputterer (HITACHI E-1030, Tokyo, Japan) and observed under a scanning electron microscope (S-4300; Hitachi, Tokyo, Japan) with an accelerating voltage of 2 kV.

### Surface roughness

Roughness measurements of the specimens before and after smoothing were performed using a profilometer (SV-624, Mitutoyo, Kanagawa, Japan). The arithmetic mean value (Ra) and the maximum height of the obtained profile, Rz, were used for the comparisons. Ra is defined as an integral of the absolute value of the roughness profile measured over the evaluation length, whereas Rz is based on the Japanese Industrial Standard (JIS B 0601:2001), and defined as the mean of the five highest peaks and lowest valleys over the sampled length.

### Pushing-force measurement of microguidewire in silicone vessel

Tensile test equipment (EZ-S, Shimadzu Corp., Kyoto, Japan) was used to measure the force necessary to propel a microguidewire through a silicone vessel. U-shaped silicone vessels, both with and without smoothing, were connected to straight silicone tubes. Inside the vascular replica, a microguidewire was inserted and the wire was held by the arm of the tester (Fig. 4a). The arm pushed the microguidewire down at a constant rate of 0.33 mm/s, and the force caused by the friction between the microguidewire and the silicone was measured. The force was measured seven times for each group.

## Additional Information

**How to cite this article**: Kaneko, N. *et al*. Manufacture of patient-specific vascular replicas for endovascular simulation using fast, low-cost method. *Sci. Rep.* **6**, 39168; doi: 10.1038/srep39168 (2016).

**Publisher's note:** Springer Nature remains neutral with regard to jurisdictional claims in published maps and institutional affiliations.
